# Correction: Beran et al. Inosine Pranobex Significantly Decreased the Case-Fatality Rate among PCR Positive Elderly with SARS-CoV-2 at Three Nursing Homes in the Czech Republic. *Pathogens* 2020, *9*, 1055

**DOI:** 10.3390/pathogens10091121

**Published:** 2021-09-02

**Authors:** Jirí Beran, Marian Špajdel, Věra Katzerová, Alena Holoušová, Jan Malyš, Jana Finger Rousková, Jiří Slíva

**Affiliations:** 1 Department for Tropical, Travel Medicine and Immunization, Institute of Postgraduate Health Education, 100 05 Prague, Czech Republic; 2 Department of Psychology, Faculty of Philosophy and Arts, Trnava University, 918 43 Trnava, Slovakia; marian.spajdel@truni.sk; 3 Domov Důchodců Litovel, 784 01 Litovel, Czech Republic; katzerova@volny.cz; 4 Sanatorium Topas, 534 01 Holice, Czech Republic; alena.holousova@sanatorium-topas.cz (A.H.); jan.malys@sanatorium-topas.cz (J.M.); 5 Senior dům Beránek Úpice, 542 32 Úpice, Czech Republic; rouskova.jana@email.cz; 6 Department of Pharmacology, Third Faculty of Medicine, Charles University, 100 00 Prague, Czech Republic; slivaj@seznam.cz

There was an error in the original article [1]. The authors wish to remove words “of this prospective trial” from the last paragraph of Section 1 (on Page 4, row 8) and delete “prospective clinical” from Section 4.1 (first sentence of this Section on Page 8). The corrected sentences are as follows: 

In last paragraph of Section 1 (on Page 4, row 8): “The aim was to critically assess the possible benefits of IP in the prevention and treatment of COVID-19 in nursing home settings.” and in Section 4.1 (first sentence of this Section on Page 8), “This study was performed in three nursing homes in three towns (Litovel, Sanatorium Topas Holice, and Beránek Úpice) in the Czech Republic”.

The authors apologize for any inconvenience caused and state that the scientific conclusions are unaffected. The original article has been updated.
